# Hysteroscopic management versus ultrasound-guided evacuation for women with first-trimester pregnancy loss, a randomised controlled trial

**DOI:** 10.1186/s12905-022-01774-2

**Published:** 2022-05-25

**Authors:** Hadeer Meshaal, Emad Salah, Eman Fawzy, Mazen Abdel-Rasheed, Ahmed Maged, Hany Saad

**Affiliations:** 1 Obstetrics and Gynecology Department, Faculty of Medicine, Cairo University, Cairo, Egypt; 2 Reproductive Health Research Department, National Research Centre, 33 El-Buhouth St, Dokki, Cairo, 12622 Egypt

**Keywords:** Missed miscarriage, Pregnancy loss, Hysteroscopy, Ultrasound-guided evacuation, Surgical evacuation

## Abstract

**Objective:**

We aimed to evaluate the hysteroscopic management of first-trimester pregnancy loss compared to surgical evacuation either blind or under ultrasonographic guidance‎.

**Methods:**

This clinical trial included ‎315 women with first-trimester pregnancy loss, divided equally into three groups. Group 1 underwent traditional blind surgical evacuation, group 2 underwent ultrasound-guided evacuation, and group 3 underwent hysteroscopic management. All women were assessed for retained products, surgical complications, the need for further management, and pregnancy occurrence after evacuation within 2 years of follow up.

**Results:**

The rate of presence of conception remnants and the need for further ‎treatment was significantly higher in group 1 compared to groups 2 and 3 (4.8% vs. 0% vs. 0%, *P* = 0.012). The conception rate within 2 years was significantly lower in group 1 compared to groups 2 and 3 (57.4% vs. 73.2% vs. 82.7%, *P* = 0.002), and the duration needed to conceive was significantly prolonged in group 1 compared to groups 2 and 3 (9.8 vs. 8.3 vs. 6.9 months, *P* < 0.001). Interestingly, women who underwent hysteroscopic management needed a significantly shorter time to conceive than those who underwent ultrasound-guided evacuation‎ (6.9 vs. 8.3 months, *P* = 0.006).

**Conclusions:**

Hysteroscopic management of first-trimester pregnancy loss was superior to ultrasound-guided surgical evacuation regarding the time interval to conceive. Both techniques were superior to the blind evacuation technique regarding removal of the whole conception remnants, need for further treatment and fertility outcomes.

*Clinical trial registration*: It was first registered at ClinicalTrials.gov on 16/03/2017 with registration number NCT03081104.

## Introduction

Missed miscarriage is defined as the retention of dead products of conception. It is one of the frustrating complications of pregnancy, and inadequacy of its management may affect not only the future ability of the female to conceive but also may be fatal. According to the World Health Organization, 67,000 women die annually due to untreated early pregnancy loss [1].

For many decades, dilatation and surgical evacuation have been considered the most common management of early pregnancy loss [2]. However, it carries the risk of many complications, most importantly the incomplete evacuation of the products of conception, resulting in intrauterine infection with subsequent intrauterine synechia and adhesions. In addition, unnecessary over curettage causes damage to the endometrium and increases the incidence of Asherman syndrome [2].

The use of prostaglandin as an adjuvant or alternative to surgery in managing early pregnancy loss became widespread. Many studies have been carried on to determine the most effective regimen and mode of administration of prostaglandins in early pregnancy loss [3, 4].

In 1973, vacuum aspiration was first used to evacuate the retained products of conception and treat early pregnancy loss [5]. Nowadays, it is widely used, being a less traumatic and simple technique in managing early pregnancy loss. The vacuum aspiration could be carried out either blindly or under ultrasonographic guidance.

The concern about the incomplete evacuation of conception products due to insufficient curettage versus the possible trauma to the endometrium that could occur due to over curettage gave rise to the idea of the evacuation of missed miscarriage under direct visualisation of the uterine cavity via operative hysteroscopy.

Our study aimed to evaluate the safety and efficacy of using operative hysteroscopy to treat early pregnancy loss compared to surgical evacuation either blind or under ultrasonographic guidance, which will be reflected in post curettage fertility rate.

## Methods

Following the CONSORT guidelines, a single-blinded randomised controlled trial was conducted at Kasr Alainy Maternity Hospital, Cairo University, between April 2017 and June 2018. The study was approved by the ethical committee of the Obstetrics and Gynaecology Department at Kasr AlAiny with registration number 263487, and registered at https://clinicaltrials.gov on 16/03/2017 with registration number NCT03081104. All participating women gave their informed consent after a full explanation of the benefits and risks of the trial by professional obstetricians.

We included 315 women with first-trimester pregnancy loss, diagnosed when the crown-rump length was 5 mm without cardiac activity or mean gestational sac diameter was 16 mm without a fetal pole and/or cardiac pulsations [6]. The inclusion criteria were maternal age between 18 and 40 years, gestational age between 6 and 14 weeks, and body mass index (BMI) less than 30. On the other hand, women with the following criteria were excluded; other types of early pregnancy loss, extrauterine gestational sac, abnormal uterine cavity, previous history of uterine surgery, moderate or severe vaginal bleeding that required immediate surgical intervention, or planning to use contraception after the operation.

All participants were subjected to full evaluation through history and examination to ensure adherence to inclusion criteria and the absence of any exclusion criteria. Transvaginal ultrasound examination was done for all participants 4 h before the procedure using a “Voluson 730” machine (GE Healthcare Austria GmbH, Seoul, South Korea) equipped with a 5–7.5 MHz transvaginal probe to confirm the diagnosis of early pregnancy loss.

On the day of the operation, women were randomised via an automated web-based randomisation system to ensure allocation concealment into three groups. All procedures were done by well-experienced senior obstetricians under general anaesthesia. After positioning the patient appropriately on the operating table, a bimanual pelvic examination was performed before the procedure to assess the axis and size of the uterus.

Women in the first group underwent traditional ‎blind uterine evacuation, i.e. without sonographic or hysteroscopic guidance. A Sims speculum was inserted into the vagina; the cervix was visualised and grasped using the Volsellum forceps. According to the gestational age, the cervical canal was dilated gradually with Hegar dilators. The uterine cavity was evacuated using a plastic cannula attached to an electric suction apparatus. The negative pressure of 75 mmHg was used. The aspirate was examined to confirm the presence of products of conception. The completeness of evacuation was checked by gentle sharp curettage and final suctioning at the end of the procedure.

In the ultrasound-guided evacuation group, the abdominal transducer was held on the abdomen to obtain a longitudinal image of the uterus and cervix and provide the surgeon with a visual reference of the gestational sac, cervical canal and any instruments passed into the uterus. The operation progress was continuously monitored as the uterine contents were evacuated under visual control. The dilators and suction cannula were kept under constant view by slightly tilting the transducer. Advancement of any instrument was allowed only under direct ultrasound control. The completeness of the evacuation in these cases was confirmed by ultrasound scanning during the procedure.

Women in the hysteroscopy guided evacuation group were put in lithotomy position after induction of anaesthesia. An expert obstetrician grasped the cervix with Pozzi forceps and dilated it to facilitate the insertion of the hysteroscope. The uterine cavity was distended with saline or glycine, depending on the polarity of the resection system, with a maximum irrigation pressure of 110 mmHg. The retained products were resected from top to bottom with a cutting loop without using the electric power. The grasping forceps was used if needed to remove any retained material. Elective coagulation cautery via hysteroscopy was done to stop intrauterine bleeding if it occurred. The deficit of distending media was calculated during the procedure.

For all three surgical procedures, vaginal misoprostol (400 mcg) was inserted 3 h before the procedure to dilate the cervix [7], and prophylactic antibiotics (Cefazolin 1 gm IV) were administered during the operation [8]. Women in the first and second groups received 5 IU of intravenous syntocinon during the procedure, while women in the third group received the dose of syntocinon immediately after the procedure [9].

The primary outcome parameter was pregnancy occurrence within 2 years after evacuation and the time needed to conceive. The secondary outcomes included operative time, the occurrence of any surgical complications during or after the procedure (such as significant bleeding, uterine perforation, presence of conception remnants in the follow-up ultrasound, and uterine infection), and the need for further management (medical treatment, or second-time surgical evacuation).

Operative time was calculated from the start of instrument introduction after cervical dilatation to the end of the procedure. Significant uterine bleeding was considered if blood loss was more than 500 ml [10]. Uterine infection (endometritis) was suspected if the temperature increased (38 °C on at least two occasions) or with the presence of abnormal vaginal discharge and pelvic tenderness.

### Sample size

Sample size calculation was done using IBM SPSS Sample Power software, release 3.0.1 (IBM Corp., Armonk, NY, USA). As considered the primary outcome, sample size calculation was done using the comparison of the proportion of pregnant women between the blind evacuation group and the other 2 groups. The calculation was done based on comparing 2 proportions from independent samples using the Chi-square test, the α-error level was fixed at 0.05 (2-tailed), and the power was set at 80%. As previously published by Hooker et al. [11], the average pregnancy rate among women who did blind surgical evacuation within variable periods (minimum 6–8 months) was 60%. The assumed minimal clinically important difference in pregnancy rate of either the hysteroscopy or ultrasound method of evacuation relative to blind evacuation was 20%. Considering about 20% dropout, the sample size was optimised to be 105 cases in each group.

### Statistical analysis

Statistical calculations were done by SPSS software, version 23 (IBM Corp., Armonk, NY, USA). The data was described in the form of mean ± SD, median (range) and count (percentages or proportions) according to data type. A comparison of means for the 3 study groups was done using the one-way analysis of variance (ANOVA) test. A comparison of proportions was done using Chi-square (χ^2^) test. Fisher test was used instead when the expected frequency was less than 5. Post-Hoc multiple comparisons were then performed. *P* values less than 0.05 were considered statistically significant.

## Results

**Fig. 1:**
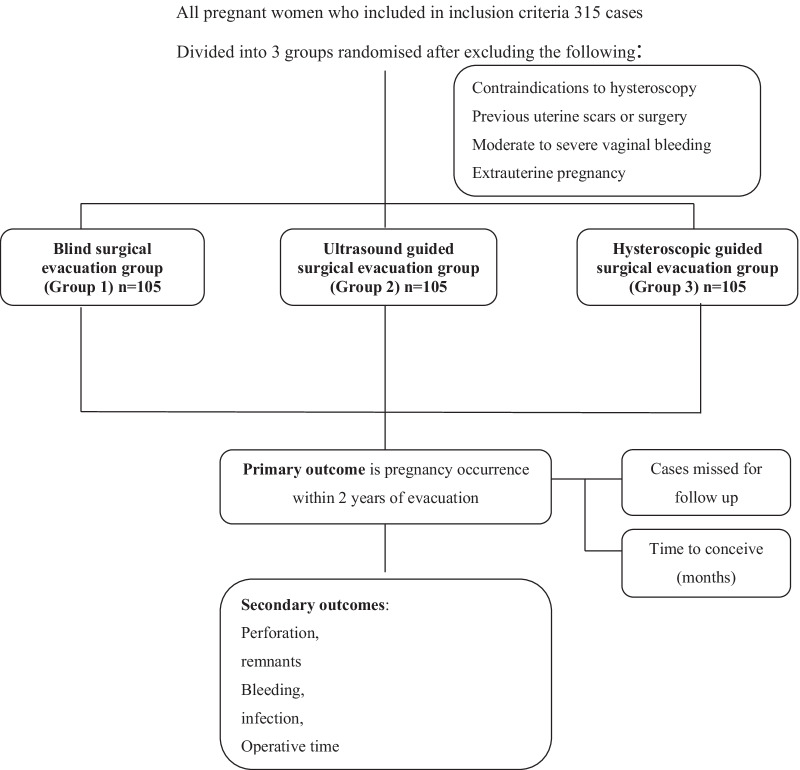
Flow of patients in the study

After excluding women who did not fulfil the inclusion criteria, 315 women were divided into three groups, as shown in the consort flow chart (Fig. 1). There was no significant difference between the three groups regarding patients’ characteristics, including age, parity, BMI and gestational age at the time of procedures (Table 1).

**Table 1:** Characteristics of the study groups

	Blind surgical evacuation group (Group 1) n = 105	Ultrasound guided surgical evacuation group (Group 2) n = 105	Hysteroscopic guided surgical evacuation group (Group 3) n = 105	*P* value
Age (years)	24.8 ± 5.9	24.9 ± 5.7	24.7 ± 5.8	0.940
Body mass index	25.9 ± 2.4	25.7 ± 2.4	25.4 ± 2.2	0.427
Parity	1 (0–3)	1 (0–3)	1 (0–2)	0.820
Gestational age at time of operation (weeks)	9.2 ± 1.2	9 ± 1.2	8.9 ± 0.9	0.296

Regarding the primary outcome measures, fewer women with the blind surgical evacuation technique (group 1) conceived within 2 years of the procedure and needed a longer time to conceive than women in the other two groups (Tables 2 and 3). When comparing women who underwent ultrasound-guided evacuation (group 2) with those who underwent hysteroscopic guided evacuation (group 3), there was no significant difference in the number of women conceived within 2 years of the procedure; however, women who underwent hysteroscopic guided evacuation need a significantly shorter time to conceive (*P* = 0.006), as shown in Table 3.

**Table 2:** Outcome measures for all groups

	Blind surgical evacuation group (Group 1) n = 105	Ultrasound guided surgical evacuation group (Group 2) n = 105	Hysteroscopic guided surgical evacuation group (Group 3) n = 105	*P* value
Operative time (minutes)	9.6 ± 3.1	9.9 ± 2.4	15.2 ± 2.4	< 0.001*
Total complications during/after operation	15 (14.3%)	7 (6.7%)	3 (2.9%)	0.010*
Presence of remnants in ultrasound follow up	5 (4.8%)	0 (0%)	0 (0%)	0.012*
Need for further treatment	5 (4.8%)	0 (0%)	0 (0%)	0.012*
Significant bleeding	6 (5.7%)	6 (5.7%)	3 (2.9%)	0.575
Uterine perforation	1 (0.9%)	0 (0%)	0 (0%)	0.990
Uterine infection	3 (2.9%)	1 (0.9%)	0 (0%)	0.329
Missed for follow up	20 (19.1%)	23 (21.9%)	24 (22.9%)	0.782
Conception rate within 2 years of follow up	49 (57.7%)	60 (73.2%)	67 (82.7%)	0.002*
Time to conceive (months)	9.8 ± 3.4	8.3 ± 2.7	6.9 ± 1.9	< 0.001*

*P value is significant

**Table 3:** Pairwise comparisons for variables having significant differences between groups

	Blind evacuation (Group 1) versus ultrasound guided (Group 2)	Blind evacuation (Group 1) versus hysteroscopic guided (Group 3)	Ultrasound guided (Group 2) versus hysteroscopic guided (Group 3)
Operative time (minutes)	− 0.33 (− 1.19 to 0.52)^a^	− 5.54 (− 6.40 to − 4.69)^a^	− 5.21 (− 6.06 to − 4.36)^a^
	NS	*P* < 0.001*	*P* < 0.001*
Total complications during or after operation	0.08 (− 0.01 to 0.16)^b^	0.11 (0.04 to 0.19)^b^	0.04 (− 0.02 to 0.09)^b^
	NS	*P* = 0.005*	NS
Presence of remnants in ultrasound follow up	0.05 (0.01 to 0.09)^b^	0.05 (0.01 to 0.09)^b^	0
	*P* = 0.030*	*P* = 0.030*	NA
Need for further treatment	0.05 (0.01 to 0.09)^b^	0.05 (0.01 to 0.09)^b^	0
	*P* = 0.030*	*P* = 0.030*	NA
Conception rate within 2 years of follow up	− 0.16 (− 0.30 to − 0.01)^b^	− 0.25 (− 0.38 to − 0.12)^b^	− 0.10 (− 0.22 to 0.03)^b^
	*P* = 0.035*	*P* < 0.001*	NS
Time to conceive (months)	1.57 (0.38 to 2.76)^a^	2.98 (1.86 to 4.09)^a^	1.40 (0.35 to 2.46)^a^
	*P* = 0.006*	*P* < 0.001*	*P* = 0.006*

*NS* not significant, *NA* not applicable

*P value is significant

^a^Tukey pairwise comparisons of ANOVA test presented as differences in means (95% CI, confidence interval)

^b^Chi-square and Fisher pairwise comparisons for proportions presented as differences in proportions (95% CI)

Upon analysing the secondary outcomes, we found that women who underwent the blind technique (group 1) significantly needed further treatment (as a result of the presence of remnants detected by ultrasound during follow up) when compared with women who underwent ultrasound-guided evacuation (group 2) and those who underwent hysteroscopic guided evacuation (group 3) (*P* = 0.030). Obviously, as shown in Table 3, there was no significant difference between groups 2 and 3 regarding the need for further treatment. The only drawback of hysteroscopic guided evacuation was the significantly long operative time compared to the blind and ultrasound-guided techniques (*P* < 0.001).

## Discussion

In cases of early pregnancy loss, surgical management is one of the treatment options [12, 13]. Surgical evacuation of early fetal demise may be done blindly, ultrasound-guided or hysteroscopic-guided. Blind evacuation of the uterus has many risks, such as uterine perforation and incomplete evacuation of the products of conception [11]. Also, the blind technique may carry the possibility of making a false passage by the dilators [14]. Nothing of these complications could happen when the uterine evacuation is done under ultrasound or hysteroscopic guidance [15, 16].

Although vacuum aspiration is currently the standard surgical management in most centres, the use of hysteroscopy has the advantage of direct visualisation of the uterine cavity in order to eliminate any risk of retained products without fear of further curettage [17, 18]. There is another advantage of hysteroscopic management over blind curettage as regards the fertility outcome, which is not only prevention of postoperative intrauterine adhesions but also hysteroscopic adhesiolysis [19].

In our study, uterine perforation occurred only in one case that underwent blind surgical evacuation, but no cases of uterine perforation were reported among the other two groups. Therefore, the use of ultrasound or hysteroscopy is supposed to reduce the rate of perforation because the cervix can be easily accessible, and uterine contents can be clearly visualised, i.e. safe removal of uterine contents without the need for further curettage. This agreed with Golan et al. [20], who found that removing retained products of conception via hysteroscopy keeps the uterine cavity intact and prevents any trauma.

Furthermore, Capmas et al. [21] found that hysteroscopic removal of retained conception products is an efficient alternative procedure as regards removing the entire uterine contents, less incidence of postoperative intrauterine adhesions and preserving the fertility rate. Our results also agreed with Acharya et al. [10], who concluded that intra-operative ultrasound is associated with a significant decrease in the complication rate of surgical management for early pregnancy loss.

Our results showed that retained products of conception were present in 5 cases who underwent blind curettage. On the contrary, no retained products were found among women of the other two groups. This agrees with the study of Caserta et al. [22], who found that ultrasound-guided evacuation reduces the incidence of incomplete curettage. In addition, Smorgick et al. [23] reported no cases of incomplete retained products of conception and then concluded that hysteroscopy is superior to blind curettage.

Our results showed a slight increase in the postoperative infection rate among the group of blind evacuation compared with the other two groups. This may be attributed to the prolonged operative time and repeated trials of introducing the curette in order to check for complete evacuation. This agrees with the study of Smorgick et al. [23], who concluded that hysteroscopy is preferable to the traditional curettage due to the lower complication rate. Also, Sotiriadis et al. [24] concluded that ultrasound-guided evacuation is more likely to induce complete uterine evacuation and is associated with less postoperative infection.

Based on our findings, the reproductive outcome was better among women who underwent ultrasound-guided evacuation and hysteroscopic management. They were able to conceive earlier, and the overall pregnancy rate was higher. Furthermore, the hysteroscopic management significantly reduced the time interval to conception compared to ultrasound-guided evacuation. In agreement with the present study, Cohen et al. [25] found a significantly reduced time interval to conception in women who underwent hysteroscopic resection compared with those who underwent blind curettage. The authors hypothesised that continuous flushing of the uterine cavity with saline solution reduces the risk of local inflammation, thus decreasing the risk of adhesion formation.

In addition, Rein et al. [17] found a statistically significant higher conception rate and shorter time to conception among the women who underwent hysteroscopy compared with those who had blind curettage. Golan et al. [20] reported that hysteroscopic removal of retained products of conception preserves the reproductive capacity. In a recent metanalysis, Vitale et al. [26] concluded that hysteroscopy can remove the retained products of conception completely in one surgical session, with lower complication rates and satisfying future fertility rate.

The main strength of our study is comparing hysteroscopic management of first-trimester pregnancy loss with both traditional blind surgical evacuation and ultrasound-guided surgical evacuation regarding the efficacy and complication rates. We also evaluated the fertility outcome along 2 years of follow up. Furthermore, we strengthened the value of ultrasound-guided surgical evacuation in non-obese women as an alternative to hysteroscopy whenever it is unavailable (for example, in limited-resources medical centres, availability of only junior physicians).

Our study had certain limitations; among these limitations was losing some patients during the follow-up period. However, there was no significant difference between the study groups regarding the number of missing cases. Another limitation is that uterine perforation could occur unnoticed in the blind surgical technique, especially with small or incomplete perforation. This fact explains why we recorded only one patient with uterine perforation among all participants.

## Conclusions

Both ultrasound-guided surgical evacuation and hysteroscopic management of first-trimester pregnancy loss are superior to blind surgical technique as regards the reproductive outcomes, presence of conception remnants, and need for further treatment. Meanwhile, women who underwent hysteroscopic management need a shorter time to conceive than those who underwent ultrasound-guided evacuation.

## Data Availability

The data that support the findings of this study are available from Kasr El-Ainy Hospital, but restrictions apply to the availability of these data, which were used under license for the current study, and so are not publicly available. Data are, however, available from the authors upon reasonable request and with permission of Kasr El-Ainy Hospital.

## References

[1] Åhman E, Shah IH (2011). Unsafe abortion: global and regional estimates of the incidence of unsafe abortion and associated mortality in 2008.

[2] Thomson AJ, Abbott JA, Deans R, Kingston A, Vancaillie TG (2009). The management of intrauterine synechiae. Curr Opin Obstet Gynecol.

[3] Kripke C (2006). Expectant management vs. surgical treatment for miscarriage. Am Fam Physician.

[4] Tanha FD, Feizi M, Shariat M (2010). Sublingual versus vaginal misoprostol for the management of missed abortion. J Obstet Gynaecol Res.

[5] Morgentaler H (1973). Report on 5641 outpatient abortions by vacuum suction curettage. Can Med Assoc J.

[6] American College of Obstetricians and Gynecologists (2018). ACOG practice bulletin no. 200 summary: early pregnancy loss. Obstet Gynecol.

[7] Ngai SW, Chan YM, Tang OS, Ho PC (1999). The use of misoprostol for pre-operative cervical dilatation prior to vacuum aspiration: a randomised trial. Hum Reprod.

[8] American College of Obstetricians and Gynecologists (2018). ACOG practice bulletin no. 195: prevention of infection after gynecologic procedures. Obstet Gynecol.

[9] Babarinsa AE, Babarinsa IA (2017). Administration of Syntocinon by anesthetists at the time of uterine evacuation in early pregnancy. J Anaesthesiol Clin Pharmacol.

[10] Acharya G, Morgan H, Paramanantham L, Fernando R (2004). A randomised controlled trial comparing surgical termination of pregnancy with and without continuous ultrasound guidance. Eur J Obstet Gynecol Reprod Biol.

[11] Hooker AB, Aydin H, Brölmann HA, Huirne JA (2016). Long-term complications and reproductive outcome after the management of retained products of conception: a systematic review. Fertil Steril.

[12] Condous G, Okaro E, Bourne T (2003). The conservative management of early pregnancy complications: a review of the literature. Ultrasound Obstet Gynecol Off J Int Soc Ultrasound Obstet Gynecol.

[13] Trinder J, Brocklehurst P, Porter R, Read M, Vyas S, Smith L (2006). Management of miscarriage: expectant, medical, or surgical? Results of randomised controlled trial (miscarriage treatment (MIST) trial). Bmj.

[14] Hooker AB, Lemmers M, Thurkow AL, Heymans MW, Opmeer BC, Brölmann HA (2014). Systematic review and meta-analysis of intrauterine adhesions after miscarriage: prevalence, risk factors and long-term reproductive outcome. Hum Reprod Update.

[15] Ben-Ami I, Melcer Y, Smorgick N, Schneider D, Pansky M, Halperin R (2014). A comparison of reproductive outcomes following hysteroscopic management versus dilatation and curettage of retained products of conception. Int J Gynecol Obstet.

[16] Haber K, Hawkins E, Levie M, Chudnoff S (2015). Hysteroscopic morcellation: review of the manufacturer and user facility device experience (MAUDE) database. J Minim Invasive Gynecol.

[17] Rein DT, Schmidt T, Hess AP, Volkmer A, Schöndorf T, Breidenbach M (2011). Hysteroscopic management of residual trophoblastic tissue is superior to ultrasound-guided curettage. J Minim Invasive Gynecol.

[18] Harpham M, Abbott J (2014). Use of a hysteroscopic morcellator to resect miscarriage in a woman with recurrent Asherman’s syndrome. J Minim Invasive Gynecol.

[19] Vitale SG, Riemma G, Carugno J, Perez-Medina T, Alonso Pacheco L, Haimovich S (2022). Postsurgical barrier strategies to avoid the recurrence of intrauterine adhesion formation after hysteroscopic adhesiolysis: a network meta-analysis of randomised controlled trials. Am J Obstet Gynecol.

[20] Golan A, Dishi M, Shalev A, Keidar R, Ginath S, Sagiv R (2011). Operative hysteroscopy to remove retained products of conception: novel treatment of an old problem. J Minim Invasive Gynecol.

[21] Capmas P, Lobersztajn A, Duminil L, Barral T, Pourcelot A-G, Fernandez H (2019). Operative hysteroscopy for retained products of conception: efficacy and subsequent fertility. J Gynecol Obstet Hum Reprod.

[22] Caserta L, Labriola D, Torella M, Di Caterina B (2008). The use of transvaginal ultrasound following voluntary interruption of pregnancy to reduce complications due to incomplete curettage. Minerva Ginecol.

[23] Smorgick N, Barel O, Fuchs N, Ben-Ami I, Pansky M, Vaknin Z (2014). Hysteroscopic management of retained products of conception: meta-analysis and literature review. Eur J Obstet Gynecol Reprod Biol.

[24] Sotiriadis A, Makrydimas G, Papatheodorou S, Ioannidis JP (2005). Expectant, medical, or surgical management of first-trimester miscarriage: a meta-analysis. Obstet Gynecol.

[25] Cohen SB, Kalter-Ferber A, Weisz BS, Zalel Y, Seidman DS, Mashiach S (2001). Hysteroscopy may be the method of choice for management of residual trophoblastic tissue. J Am Assoc Gynecol Laparosc.

[26] Vitale SG, Parry JP, Carugno J, Cholkeri-Singh A, Della Corte L, Cianci S (2021). Surgical and reproductive outcomes after hysteroscopic removal of retained products of conception: a systematic review and meta-analysis. J Minim Invasive Gynecol.

